# Editorial: Case reports in pediatric hematology and hematological malignancies 2022

**DOI:** 10.3389/fped.2023.1254343

**Published:** 2023-07-28

**Authors:** Paulo S. S. Santos, Hasan Hashem, Daniele Zama

**Affiliations:** ^1^ Department of Surgery, Stomatology, Pathology and Radiology, Bauru School of Dentistry, University of São Paulo, Sao Paulo, Brazil; ^2^ Division of Pediatric Hematology Oncology and Bone Marrow Transplantation, Department of Pediatrics, King Hussein Cancer Center, Amman, Jordan; ^3^ Department of Medical and Surgical Sciences, Alma Mater Studiorum, University of Bologna, Bologna, Italy; ^4^ Pediatric Emergency Unit, IRCCS Azienda Ospedaliero-Universitaria di Bologna, Bologna, Italy

**Keywords:** children, onchology, hematology, immunology, stem cell transplantation (HSCT)

**Editorial on the Research Topic** Case reports in pediatric hematology and hematological malignancies 2022

Case reports on pediatric hematology and hematologic malignancies are featured in this issue. Oncohematology and pediatric hematology are poorly characterized in the scientific literature, particularly in case reports. This type of information, based on professional experience with clinical cases, is very relevant for clinical practice because it enables the professional who attends to this group of patients to adequately clarify and manage these cases, bringing the appropriate resolution to many cases and improving the quality of life of these patients.

The case described by Thalji et al. included a 4-year-old male boy who had a blueberry muffin rash on his face and neck but had no additional systemic involvement. A biopsy revealed that the patient had congenital cutaneous LCH. This early detection shields patients from needless and potentially harmful systemic treatment (Thalji et al.).

The case reported by Wang et al. reported a 13-year-old male child with recurrent epistaxis for 6 months, anemia for 1 month, and a chest CT scan compatible with hemorrhagic foci was given in the second case report. He was diagnosed with acquired von Willebrand syndrome, had no family history, and tests revealed that he had antinuclear and anti-Sm antibodies, indicating a link to Systemic Lupus Erythematosus (Wang et al.).

The third case, described by Jenei et al., is a 5-year-old male child diagnosed with interdigitating dendritic cell sarcoma during treatment for B-cell precursor acute lymphoblastic leukemia, according to another clinical case described in this issue. Multiple lymph nodes in the cervical chain were discovered in this patient, who was detected with positron emission tomography CT, treated with chemotherapy, and then had bone infiltration 4 years later. With this condition, an allogeneic hematopoietic stem cell transplant was conducted successfully (Jenei et al.).

Zhou et al. described a 6-year-old girl who had a clinical manifestation of leukemia cutis with a 10-month history of presentation with cutaneous plaques and abscesses as furuncles. The initial clinical indications of Chronic Neutrophil Leukemia BCR-ABL Negative Myeloproliferative Neoplasm were these cutaneous appearances. Successful allogeneic hematopoietic stem cell transplantation was also used to treat the patient. This discovery was pertinent to dermatologists (Zhou et al.).

A report of Hannemann et al. highlighted a major but rare complications of allogeneic hematopoietic cell transplantation (HCT): an invasive fungal infection with fusariosis as a lethal infections despite neutrophil engraftment and uninterrupted treatment with voriconazole. Unfortunately, this 16-year old patient with severe aplastic anemia developed splenic rupture and endocarditis. He also developed embolic cerebral infarctions with unilateral hemiparesis, and despite cardiac surgery, he did not regain consciousness because of diffuse cerebral ischemia and died on day +92 post HCT. Disseminated infection with fusarium solani is a rare complications post allogeneic HCT with poor outcomes (Hannemann et al.).

The case presented by Krstic et al. described the first case of T-cell acute lymphoblastic leukemia (T-ALL) with CCDC88C:JAK2 fusion, with normal karyotype, detected by whole genome sequencing. The patient fortunately, responded to nelarabine and intensive chemotherapy and so proceeded to allogeneic HCT. The patient is now in remission 8 months post HCT. High throughput sequencing technologies have shown enormous potential in the genetic characterization of hematological malignancies, particularly in pediatric ALL, and are paving the way to personalized treatment strategies. Pediatric patients with ALL and genomic features consistent with JAK/STAT pathway activation are currently recruited in ongoing clinical trials for targeted treatment with Ruxolitinib in addition to chemotherapy. However, this report serves as an example of the power of WGS in the diagnostic setting of acute leukemia, as it enables timely recognition of potential therapeutic targets in high-risk pediatric ALL (Krstic et al.).

The case presented by Ipe et al., reported a 12-year old patient with B-ALL who relapsed 15 months after diagnosis while receiving maintenance chemotherapy. For his relapsed disease, he underwent CAR-T cell therapy to avoid allogeneic HCT and risk of DNA damage with radiation due to lately discovered germline heterozygous checkpoint kinase 2 (CHEK2) mutation. This mutation was discovered when the patient presented with papillary thyroid carcinoma 7 months after initial diagnosis and before relapse of his B-ALL. The patients remains in remission 3 years after CAR-T cell therapy. CHEK2 is a tumor suppressor gene that plays a critical role in the cell cycle and response to DNA damage induced by replication stress. Whether the CHEK2 gene is a true cancer predisposition syndrome gene remains a topic of debate especially in relation to acute leukemia. More research in delineating the true role of pathogenic CHEK2 gene mutations in hematologic malignancies as a potential cancer predisposition gene is needed (Ipe et al.).

In the report of Leonardi et al., a patient with a long and really challenging clinical history is reported. Kabuki syndrome (KS) is a rare multisystemic disease due to mutations in the *KMT2D* or *KDM6A* genes, which act as epigenetic modulators of different processes, including immune response. The syndrome is characterized by anomalies in multiple organ systems, and it is associated with autoimmune and inflammatory disorders, and an underlying immunological phenotype characterized by immunodeficiency and immune dysregulation. This case emphasizes the importance of suspecting immune dysregulation in KS (Leonardi et al.).

In the report of Naviglio et al., a case of treatment with venetoclax and azacitidine in a patient with AML in Shwachman-Diamond syndrome is reported. BCL-2 inhibitor venetoclax has revolutionized the treatment of AML in elderly adults, especially for treatment-naive elderly patients who are ineligible for intensive chemotherapy and there is limited evidence on the use of venetoclax in pediatric patients with SDS-related MDS or AML. This represents an experience suggestisting the potential role of the combination venetoclax/azacitidine for patients with SDS and AML, whose safety and efficacy need to be evaluated in a clinical experimental model (Naviglio et al.).

In the report of Akiely et al. the first pediatric case of myeloid/lymphoid neoplasm with PDGFRA rearrangement presenting with synchronous myeloproliferative disease and B-LBL. The patient was started on imatinib with concomitant therapy for B-LBL per the Children Oncology Group (COG) standard therapy for localized B-LBL and demonstrated a favorable outcome in the 2.5-year follow-up period (Akiely et al.).

In this report Guerra et al. describe a rare case of patients with pediatric myelofibrosis, showing different clinical and pathological features when compared to the WHO 2016 Primary Myelofibrosis classification. They aslo retrospectively collected and analyzed 14 consecutive pediatric myelofibrosis, classified into three subgroups: adult-like myelofibrosis, pediatric immune myelofibrosis, idiopathic myelofibrosis. Pediatric Immune Myelofibrosis was the predominant subgroup in our cohort (7/14). Pediatric Immune Myelofibrosis is characterized by peculiar bone marrow features (i.e., T lymphocyte infiltration) and a milder course compared to the other patients Pediatric Immune Myelofibrosis is a novel and distinct pathological entity (Guerra et al.).

In the report Eguchi et al. describe 10-month-old infant with an extremely rare combination of Juvenile Myelomonocytic Leukemia and Ornithine Transcarbamylase Deficiency Safely. One noteworthy aspect of the present case is the proven involvement of congenital UCD in the pathogenesis of hyperammonemia associated with cancer treatment. The treatment of the patient was complicated by occurrence of severe hyperammonia. Authors underline that hyperammonemia should be considered a differential diagnosis when unexplained and non-specific symptoms occur during the treatment of hematologic malignancies (Eguchi et al.).

In the report of Saettini et al. two patients with identical germline *CBL* mutation and clinical and immune-hematological overlapping features with autoimmune lymphoproliferative syndrome (ALPS) and B-cell expansion with NF-κB and T-cell anergy (BENTA) syndrome are described. CBL syndrome is a Noonan-like RASopathy with heterogeneous clinical phenotype and predisposition to juvenile myelomonocytic leukemia (JMML). Although the phenotype of children affected by CBL syndrome has been increasingly delineated over time, the immunological features of CBL syndrome have not been extensively described (Saettini et al.).

We really hope that reading this issue, which contains these rare clinical situations in pediatrics, hematology, and oncohematology, would be beneficial to professional knowledge and clinical care for this population.

